# Allergy and autoimmunity in children: non-mutually exclusive diseases. A narrative review

**DOI:** 10.3389/fped.2023.1239365

**Published:** 2023-11-02

**Authors:** Enza D’Auria, Martina Minutoli, Alessandra Colombo, Marco Ugo Andrea Sartorio, Fiammetta Zunica, Gianvincenzo Zuccotti, Vassilios Lougaris

**Affiliations:** ^1^ Department of Pediatrics, Vittore Buzzi Children’s Hospital, University of Milan, Milan, Italy; ^2^ Department of Pediatrics, Fatebenefratelli Hospital, Milan, Italy; ^3^ Department of Biomedical and Clinical Sciences, University of Milan, Milan, Italy; ^4^ Department of Clinical and Experimental Sciences, ASST – Spedali Civili di Brescia, Paediatrics Clinic and Institute for Molecular Medicine A. Nocivelli, University of Brescia, Brescia, Italy

**Keywords:** pediatrics, children, allergy, autoimmunity, atopic dermatitis, asthma, celiac disease, type 1 diabetes mellitus

## Abstract

In last decades a simultaneous increase in the prevalence of atopic and autoimmune disorders in pediatric population has been observed. Despite the Th1-Th2 paradigm, supporting the polarization of the immune system with Th1 response involved in autoimmune diseases and Th2 response leading to hypersensitivity reactions, recent evidence suggests a possible coexistence of common pathogenic pathways as result of shared immune dysregulation. Similar genes and other mechanisms such as epithelial barrier damage, gut microbiota dysbiosis and reduced number of T regs and IL-10 contribute to the onset of allergy and autoimmunity. IgA deficiency is also hypothesized to be the crosslink between celiac disease and allergy by lowering gut mucous membrane protection from antigens and allergens. The present narrative review aims to give an overview of the co-occurrence of allergic and autoimmune disorders (celiac disease, inflammatory bowel diseases, type 1 diabetes mellitus, thyroid disease, juvenile idiopathic arthritis) in pediatric population, based on the available evidence. We also highlighted the common pathogenic pathways that may underpin both. Our findings confirm that allergic and autoimmune diseases are commonly associated, and clinicians should therefore be aware of the possible coexistence of these conditions in order to ameliorate disease management and patient care. Particular attention should be paid to the association between atopic dermatitis or asthma and celiac disease or type 1 diabetes and vice versa, for therapeutic interventions. Further studies are needed to better clarify mechanisms involved in the pathogenesis and eventually identify new therapeutic strategies.

## Introduction

For more than two decades the Th1-Th2 paradigm supported the polarization of the immune system: according to this theory, internal and external factors act together to induce either a Th1 or a Th2-mediated response defining the balance between these two different inflammatory patterns (1).

**Figure 1:**
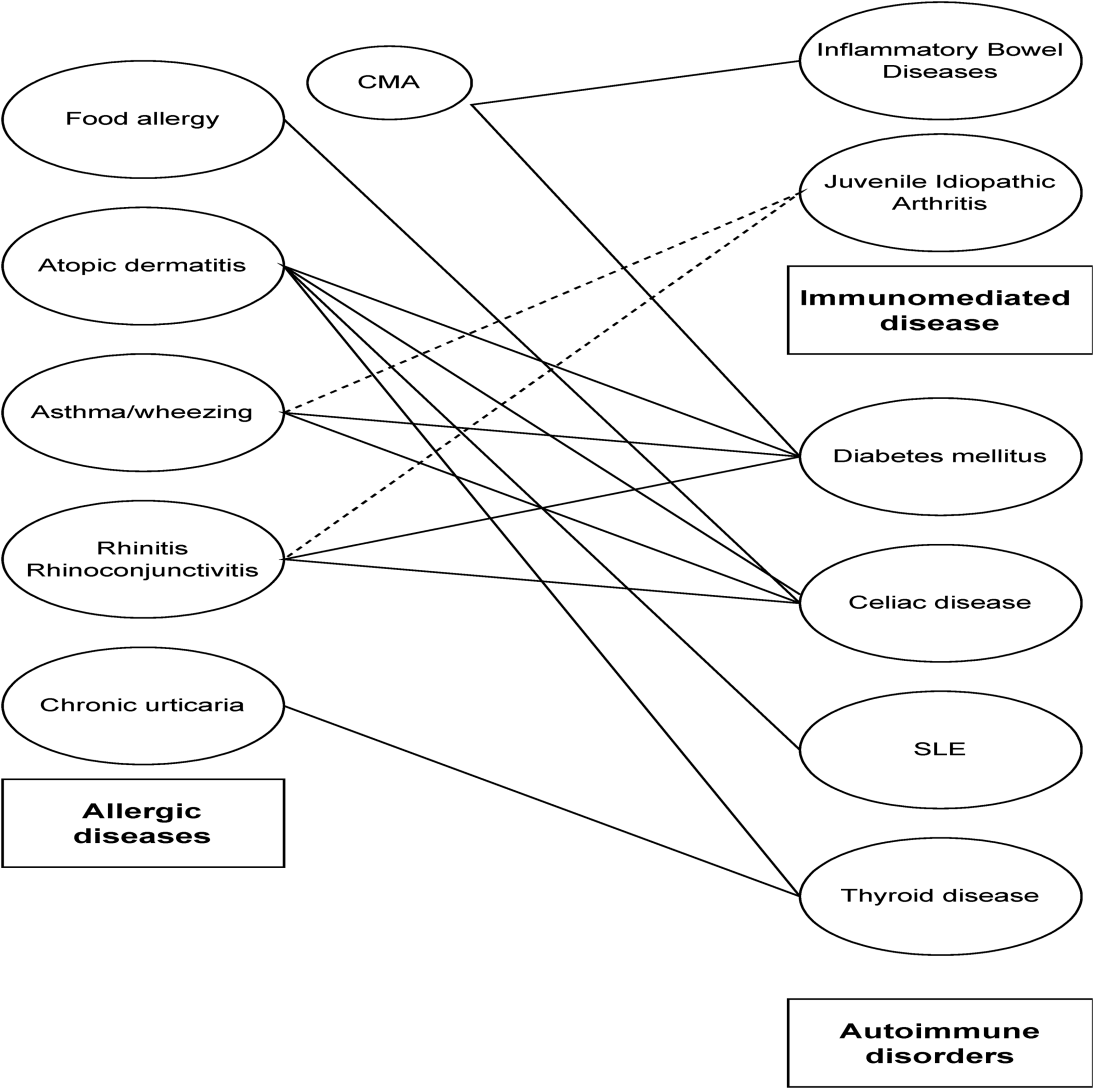
Association between allergic, autoimmune and immune-mediated disorders. Solid lines refer to association between diseases, while dotted lines suggest weak association as highlighted in the main text.

The Th1-response is mediated by the release of proinflammatory cytokines such as interferon-*γ*, IL-2 and lymphotoxin-α, which leads to B-cell production of IgG antibodies, macrophageactivation, cell cytotoxicity, and induction of cellular immunity; an abnormal activation of this pathway may lead to autoimmune phenomena. On the other hand, TH2 cells produce cytokines such as IL-4, IL-5, IL-13, promote eosinophils’ activation, induce antibody class-switching to IgE and are implicated in allergic-type hypersensitivity reactions (2).

However, other cells, such as macrophages and epithelial cells have been shown to have a similar cytokine production pattern, thus making even more complex the pathogenetic mechanism of allergic diseases (3).

A recently identified cell population, the ILCs (Helper innate lymphoid cells), play a fundamental role in the early immune response and therefore in the homeostasis of the immune system. They are a source of cytokines whose phenotype is matched by the adaptive system. In particular, ILC2s are involved in allergic diseases of the respiratory system as well as TH2 cells (4) and promote the expansion of IL10-producing T-regs (5). ILC2s are activated in allergic conditions such as asthma, allergic rhinosinusitis and atopic dermatitis (5). on the other hand, INF-gamma-producing ILC1s, via the ILC1/alarm axis, seem to play a central role in the pathogenesis of inflammation in autoimmune diseases, including celiac disease (6).

Thus, understanding their functions is necessary to deeply understand the pathogenetic basis of allergic manifestations and autoimmune diseases.

In recent decades there has been a significant increase in the incidence of allergic disorders, reaching almost 20% of high-income populations (7–9), In parallel, an increasing trend of autoimmune disorders has been recorded in children living in developed countries (10–13) with an estimated prevalence ranging between 7.6 and 9.4% worldwide (14).

This simultaneous increase in the prevalence of atopic and autoimmune disorders has supported the hypothesis of a common etiopathogenetic background, although the exact pathogenetic pathways are far to be understood (15).

The major hypothesis to support this theory is currently the hygiene hypothesis, proposed in 1989 by Strachan (16). It suggests that the increased incidence of allergic and autoimmune diseases has been a result of a reduced infectious pressure given to the modern Western lifestyle: the exposure to certain infectious agents early in life seems to play a protective role against inflammatory diseases and its significant progressive reduction over the last decades, due to hygiene measures, has resulted in the immune system's dysregulation and in the development of allergy and autoimmunity.

Recently, it has been postulated that epithelial barrier damage (the so called “barrier hypothesis”) caused by different environmental factors, such as agents of industrial and urban environment, may drive the transepithelial translocation of pathogenic opportunistic microbes, promoting inflammatory processes and eliciting immune dysregulation (17).

For instance, substances as laundry detergents and household cleaning agents have widely been shown a directly disruptive effects on the tight junctions barrier integrity of epithelial cells (18), as well prolonged exposures to cigarettes smoke and air pollutants have been demonstrated to result not only in lung epithelium damage, but also in immune system impairment through release of proinflammatory cytokines and chemokines, enhanced recruitment of macrophages and neutrophils and increased Th2-dependent responses (19).

Of note, epithelial barrier leakiness has been reported in several autoimmune diseases, e.g., celiac disease, IBD, eosinophilic esophagitis, diabetes mellitus type 1, rheumatoid arthritis, systemic lupus erythematous and atopic disorders (AR, AA, AD and FA) (20–22).

Recent studies have also highlighted the effect of diet on gut microbiota composition and the connection to immunological pathways. The insufficient intake of ‘‘healthy foods” in Western dietary habits adversely affects the production of bacterial metabolites which are crucial for regulation of inflammatory response (23). Furthermore, the consumption of processed food containing additives and preservatives increases intestinal permeability to allergens and pathogens (24).

In recent years, the B regs have assumed an important role in the understanding of immune mechanisms: they perform regulatory functions in inflammatory conditions with an immunosuppressive effect and maintaining tolerance, re-establishing the immune homeostasis. Recent studies have shown that in chronic inflammatory conditions such as autoimmune and allergic diseases the number of circulating B regs and the production of IL10 are reduced. IL-10 has a protective role in allergic inflammation and can inhibit TH2 polarization and Th17 mediated responses (25, 26).

The complex mechanism of tolerance is maintained thanks to a complex network between several cell types, such as T and B lymphocytes, dendritic cells (DC) and others. Alterations in DC migration can lead to their abnormal activation, resulting in imbalance of immune responses that may eventually contribute to the onset of autoimmune manifestations, infectious and allergic diseases, as well as cancers (27). Furthermore, the chronic inflammatory state may induce the formation of neo-epitopes that escape central tolerance and promote the formation of autoantigens with massive activation of self-reactive T lymphocytes (28). The loss of certain microbial species in gut microbiota, in the presence of reduced microbiota diversity and dysbiosis characteristic of different chronic non communicable diseases, plays a crucial role in tolerance disruption (29, 30).

These environmental factors add up to a genetic predisposition for immunity impairment. Studies on the human genome have revealed a large number of genetic factors implicated in the origin of atopic and autoimmune conditions, such as HLA haplotypes, genes encoding cytokines or their receptors (31).

The review’s purpose is to present an overview of the existing evidence supporting the co-occurrence of allergy and autoimmunity in pediatric population.

## Methods

### Search strategy

A comprehensive search on Medline via PubMed and EMBASE (from January 1, 2000, through June 30, 2022), restricted to pediatric age by using the medical subject heading terms referring to atopic disease (“atopy”, “allergy”, “food allergy”, “asthma”, “allergic rhinitis”, “atopic dermatitis”, “urticaria”) each one combined with terms referring to autoimmune disease (“celiac disease”, “inflammatory bowel disease”, “Crohn’s disease”, “ulcerative colitis”, “diabetes mellitus”, “type-1 diabetes mellitus”, “thyroid autoimmune disease”,“Hashimoto disease”, “Juvenile Idiopathic Arthritis”) building search strings with Boolean operators “AND”.

We restricted our search to English-language publications. We did not restrict for type or study design. Duplicates found between searches were identified and removed. Studies were excluded if the information was not specific to the topic of this review.

### Celiac disease and allergic disorders

Celiac disease (CD) is a rather common chronic autoimmune disease involving the gastrointestinal tract in genetically predisposed subjects, affecting about 1% of the general population (32), and up to 3% of some Western pediatric populations (33).

Several studies have investigated the association between CD and allergic disorders, both in adults and children.

A higher incidence of CD in subjects suffering from allergic diseases (e.g., asthma, AD, allergic rhinitis/conjunctivitis) has been observed in most observational studies (33–38).

Narla et al.'s study found that AD was correlated to several autoimmune disorders in children, particularly alopecia areata, vitiligo, scleroderma, and chronic urticaria; however, when examining specifically the correlation between AD and CD in pediatric populations, this correlation did not result significant (39).

The meta-analysis by Lu et al. showed a significant association of atopic dermatitis with multiple autoimmune diseases, including celiac disease (40).

In parallel, the frequency of AD has been also found to be significantly higher in children with CD respect to general population, pointing to a bidirectional link between these diseases (35, 37).

Concerning food allergy (FA), existing data are still controversial.

IgE-mediated FA was found to be more common in children affected by CD, and CD prevalence resulted to be higher in the occurrence of severe FA compared to the general pediatric population and subjects with mild forms of allergy (37, 41). Conversely, Lanzarin et al. did not find a significant increase in sensitization to wheat, rye, barley, and malt in children with CD (42).

The relationship between CD and asthma has also been investigated (35, 43–47) in pediatric populations.

Kero et al. found a significantly higher incidence of asthma in children with CD than in controls (43) and this correlation seemed to be present both before and after CD diagnosis (45).

A Swedish study also showed an increased incidence of celiac disease in asthmatic patients, but only in young age (44).

Similarly, Canova et al. found asthmatic children to have an increased risk of developing CD (46). Of note, a retrospective study conducted in the USA confirmed the association between asthma and CD only in a subgroup of children who also had a positive family history for asthma (47).

Table 1 summarizes the studies concerning the association between CD and allergic disorders.

**Table 1:** Studies investigating the association between CD and atopic diseases.

First author and country	Year	Study design	Study group	Atopic disorder	Results	*P* value	Ref
Lu Z et al. (Taiwan)	2021	Metanalysis of cross-sectional or case–control studies	90,568,121 patients with AD	Atopic dermatitis	Higher prevalence of CD in AD patients in 4 studies, with an average OR of 1.98 [1.51–2.60]. Increased incidence of CD in AD patients in one cohort (RR 1.41 [1.32–1.50])	*p* = 0.000	(40)
Shalom et al. (Israel)	2020	Cross-sectional study	Population-based (116.816 subjects)	Atopic dermatitis	Significant correlation between AD and CD in both adults and children (pediatric patients with AD vs. pediatric subjects without AD: OR 1.565 [1.34–1.87])	*p* < 0.001	(38)
Krishna et al. (UK)	2019	Longitudinal retrospective cohort-study	Population-based—patients from UK primary-care database (1.393.570 subjects)	Atopic dermatitis; asthma; allergic rhinitis/conjunctivitis	Higher incidence of autoimmune pathologies, including CD, in subjects suffering from asthma (aIRR 1.44 [1.34–1.55]), AD(aIRR 1.41 [1.32–1.50]), and ARC (aIRR 1.39 [1.28–1.51]) compared to controls.	N/A	(34)
Ress et al. (Estonia)	2014	Case-control study	School aged children	Atopic dermatitis	1.4% prevalence of CD in Estonian children with AD, which was 4 times higher (OR 4.18, [1.12–15.64]) than controls	N/A	(33)
Kauppi et al. (Finland)	2021	Retrospective case-control study	Pediatric population present in the Finnish Care Register for Health and Care	Atopic dermatitis	Higher prevalence of CD in children with AD (OR 2.28 [2.07–2.52]	N/A	(36)
Yavuzyilmaz et al. (Turkey)	2019	Cross-sectional study (questionnaire-based)	Children aged 8–18 years	Atopic dermatitis	Higher prevalence of AD in children with CD	*p* = 0.039	(35)
Cudowska et al.	2021	Retrospective study	59 children hospitalized for CD between 2016 and 2018	Atopic dermatitis; food allergy	Higher prevalence of AD in children with CD + IgE-mediated sensitization (33.3% vs. 14.9%)	*p* > 0.05 (not statistically significant)	(37)
Pillon et al.	2015	Case-control study	319 children aged 5–13 years	Food allergy	CD prevalence in patients with food allergy resulted to be 4–5 times higher than general population and subjects with mild forms of allergy	*p* < 0.0001 (healthy school children) *p* = 0.03 (children w/ mild allergy)	(41)
Lanzarin et al. (Brazil)	2020	Descriptive cross-sectional study	Patients aged 1–20 years old diagnosed with CD from a Pediatric Gastroenterology celiac disease Clinic of São Paulo	Food allergy	The frequency of food sensitization in CD patients was not found to be higher than in the general population	Not significant	(42)
Kero et al. (Finland)	2001	Retrospective cohort study	Children born in 1987 (Finnish Medical Birth Register) followed-up for first 7 years of life	Asthma	Cumulative incidence of asthma in a pediatric population significantly higher in children with CD than in controls (24.6% and 3.4%,)	*p* < 0.001	(43)
Hemminkiet al. (Sweden)	2009	Retrospective cohort study	Asthmatic patients registered in the Hospital Discharge Register of Sweden	Asthma	The incidence of autoimmune diseases in asthmatic patients revealed that some autoimmune diseases, including CD, were of increased incidence only in young asthmatic patients (IR 1.42 [1.09–1.80])	N/A	(44)
Ludvigsson et al. (Sweden)	2011	Retrospective cohort study	Patients with CD and controls	Asthma	Positive association between asthma and CD (HR 1.61[ 1.50–1.72]), both before (OR 1.44 [1.34–1.56])and after (HR 1.42 [1.09 1.80]) CD diagnosis	*p* < 0.001	(45)
Canova et al. (Italy)	2015	Longitudinal cohort study	Registry-based population birth cohort followed-up for the first 17 years of life	Asthma	Asthmatic children were found to have an increased risk of developing CD (IRR 1.46, 95% CI 1.25–1.67)	*p* < 0.001	(46)
Patel et al. (USA)	2018	Retrospective case-control study	Children aged 6–13 years	Asthma + family history	Confirmed the association between asthma and CD only in a subgroup of children who also had a positive family history for asthma (OR 2.8 [1.3–6.0])	*p* = 0.008	(47)

AD, atopic dermatitis; CD, celiac disease; ARC, allergic rhinitis and conjunctivitis, aIRR, adjusted incidence rate ratios; IRR, incident rate ratios; IR, incidence ratios; OR, odd ratios; HR hazard ratios.

The exact mechanisms linking CD to allergic disorders are still unknown. A reduced microbial exposure and altered intestinal microbiota, caused by genetic and multiple environmental factors underpin common immune dysregulation, both antibodies-mediated and tissue-mediated (43). The crucial role of gut dysbiosis is supported by the fact that children exposed to antibiotics in early life seem to be at increased risk of developing childhood-onset asthma, allergic rhinitis, AD, and CD. Furthermore, multiple prescriptions appear to predispose to the coexistence of multiple conditions (48).

A poor vitamin D status has also been considered as a possible factor contributing to the development of both allergic and autoimmune diseases, as more than half of CD patients present reduced levels of 25-(OH) vitamin D several years after being diagnosed with this disorder (49).

Furthermore, a vitamin D deficiency is inversely proportional to the increase in ILCP cells involved in tissue inflammation at the level of the duodenal mucosa (50) Vitamin D has anti-inflammatory properties by reducing the release of IFN-gamma from (51). Low levels of vitamin D reduce the effect of regulatory T cells (T-regs), causing ineffective control of T-cell mediated responses, which could lead to the development of asthma. On top of this, shared genetic factors and excessive oxidative stress might also be possible mechanisms linking CD and asthma (45).

In regard to the co-occurrence of CD and FA, a damaged epithelial barrier and an impaired intestinal permeability might represent a possible causative link (37, 41).

ILC1-induced inflammation, mediated by IFN-gamma, contributes to barrier damage which in turn induces the release of alarmins, which cause loss of tolerance of gluten-derived peptides (6).

Some authors speculate whether elimination of foods from diet, as gluten-free diet, could result in loss of tolerance, favoring IgE-mediated reactions (52–54). Furthermore, chronic up-regulation of IL-15 in the intestinal mucosa, a typical finding in CD, might be responsible for the dysregulation of several immune mechanisms, resulting in Th1- and Th2-related disorders (55, 56). Finally, dysfunction of the epidermal barrier, altered microbiota, and immune dysregulation might play a role the mechanisms linking CD and AD (37, 40).

*I*gA deficiency (IgAD) deserves a specific attention. It is the most common primary immunodeficiency strongly associated with an increased predisposition to develop allergic and autoimmune disorders such as celiac disease with a risk of about 10–20 times. Although the pathogenetic factors of the aforementioned association are not yet fully known, many studies have described it in the pertinent literature (57).

Janzi et al. measured the serum IgA levels in 2423 children of 52 months of age, 14 with IgAD and 2,409 without IgAD, observing that subjects with low levels of IgA have a major prevalence non-IgE mediated food allergy (58).

Aghamohammadi et al. studied a sample of 37 patients with IgA deficiency, aged between 4 and 32 years, in which the onset of disease was represented by allergic conditions. Interestingly, the 25% of IgAD have been diagnosed during an allergologic assessment. Likewise, autoimmune diseases were found in 10 patients of which 4 affected by autoimmune thyroiditis (59).

Odineal et al. suggest to pay extra attention to patients with IgAD considering its strong association with the autoimmune diseases—e.g., SLE, dysthyroidism, JIA, vitiligo (60).

Aytekin et al. confirmed the aforementioned results, demonstrating that allergic diseases often represent the second, clinical manifestation in patients with IgAD in 43.2% of cases; likewise, the prevalence of autoimmune conditions is 3%–5% with a diagnosis of celiac disease in 4 patients (61).

A single-center study evaluated the clinical features of 184 patients with IgA deficiency demonstrating a high occurrence of allergic manifestations and celiac disease, respectively 39% and 14% (62). A retrospective cohort study, including both adult and pediatric populations, showed a prevalence of 2.06% and 1.89% of celiac disease in patients with selective IgA deficiency and partial IgA deficiency respectively, without significant differences sex and age related (63).

Recent studies support the association between IgA deficiency and allergy with good prognosis, although the possible association with autoimmune features and recurrent respiratory infections may have different clinical outcomes (64).

Cinicola et al. showed that patients with IgA deficiency and allergies do not have a complex immune defect, except for transient mild lymphopenia and low count CD19 + at diagnosis vs. follow-up (65% vs. 1.5%, *p* < 0.0001% and 57% vs. 11%, *p* < 0.0001, respectively) (65).

The salivary IgA seems to play a pivotal role in the protection of the mucous membranes by hindering the entry of allergens (66). In this sense, the authors speculate that the IgAD may be involved in the development of sensitization (66). Of note, the association between the IgAD and autoimmune diseases may also be related to a common genetic background—e.g., the TNFRSF13B gene encodings TACI (67). Lastly, it has been demonstrated that the HLA 8.1 haplotype is a genetic marker characterizing both autoimmune diseases and IgA deficiency (68).

To date, it is not possible to point out a clear association between celiac disease and allergies, but there is increasing evidence on emerging role of ILC at the core of inflammatory pathways both in autoimmune and allergic diseases, which mayexplain the coexistence in the same patient of these disorders. Furthermore, many studies, although a heterogeneity of the prevalence estimates, confirm the direct association existing between IgA deficiency and allergic diseases.

### Intestinal bowel diseases and allergic disorders

Ulcerative colitis and Crohn disease are chronic inflammatory disorders of the gastrointestinal tract with a rising incidence in pediatric populations (about 25% of patients with IBD receive diagnosis before the age of 20 years). IBDs are classically defined as chronic inflammatory immune-mediated disorders (69). Many genetics and external factors, such as intestinal bacterial antigens, can indeed alter host's mucosal barrier function and trigger inappropriate and ongoing activation of both humoral and cell-mediated mucosal immune system (70, 71). However, autoimmune phenomena also take place in IBD pathogenesis, resulting in antibodies and autoantibodies production, such as anti-neutrophil cytoplasm antibodies (ANCA) and anti-Saccharomyces cerevisiae antibodies (ASCA), although their pathogenic role has not been fully clarified (69).

The disruption of the balance between Th17 and Treg underlies the pathogenesis of IBD. Th17 cells infiltrate the intestinal mucosa and release a greater amount of IL17 with a proinflammatory action. At the same time, Tregs decrease and lose their inhibitory action (72). It was also shown that genetic susceptibility plays an important role in pediatric, but not adult, onset of IBD (73).

Most studies found a positive correlation between AD and autoimmune disease of gut mucosa, as Crohn's disease and Ulcerative colitis (40, 74–76). In addition, there is also evidence, both in pediatric and adult population, of a bidirectional association between atopic dermatitis and inflammatory bowel diseases, which could be explained from a common genetic background*:* in fact, impaired expression of genes such as *ILR6, IL1RL1, IL18R1, IL18RAP* was detected in AD as well as in IBD patients (40, 77).

Specific IgE to food allergens were detected in serum of children with IBD (78), although this phenomenon could be related to IL-5 release from overactivated mast cells in inflamed mucosa (78, 79). Nevertheless, there is evidence of clinical coexistence of food allergy and IBD. A significantly increased risk of developing pediatric-onset IBD was observed in children with CMA in infancy (8.2% among patients with CD, 6.4% in UC, 4.0% among the controls). Besides, children with a diagnosis of CMA seemed to contract PIBD at a younger age than the respective non-CMA group (80, 81).

Furthermore, an increased prevalence of asthma, eczema, and allergic rhinitis was found in children who suffered from IBD (82). A population-based study confirmed an increased risk of developing IBD in asthma-affected patients, without any influence of the age at diagnosis for Crohn's disease. Of note, a significant association of asthma with UC was observed only in individuals diagnosed with asthma before age 17 (83). The risk of asthma seems also to diminish with the increase of IBD onset age: VEO-IBD children accounts for the highest risks of developing asthma compared to a progressively lower risk in EO-IBD and pediatric IBD (84, 85).

The possible underlying etiopathogenetic mechanism linking atopy to IBD is still under discussion. One of the IBD hallmarks is a defective mucosal barrier with decreased or altered tight junctions (86). Microbiota alterations play a key role, since it has been demonstrated that intestinal Th17 cells increases and induced colonic inflammation in germ-free mice by transferring gut microbes from IBD mice (87–89). Gut microbes promote naive CD4+ T cells differentiation into Th17 cells by metabolites and direct induction; the upregulation of TH 17 contributes to the recall of cells and production of inflammatory cytokines, e.g IL-6, IL-1b and IL-23. IL-6, IL-22 (90).

Existing evidence has suggested that the gut microbiota can induce Th17 cell differentiation either directly through contact with immune cells or through metabolites indirectly.

In recent years, more data was published regarding the pathogenetic role of IL-17 in some subgroups of allergic diseases, although the involvement of the Th17 cytokine in the pathophysiological pathway is not conclusively understood (91, 92).

In clinical practice, an increasing occurrence of AD, food allergy and asthma in children with inflammatory bowel diseases has been observed, probably due to alteration of the common alteration of the barrier function and pivotal role of IL17.

### Type 1 diabetes mellitus and allergic disorders

Type 1 diabetes mellitus is an autoimmune disorder typically affecting children. The incidence of type 1 diabetes mellitus in childhood and adolescence is steadily rising and now stands at 22.9 new cases per year per 100,000 persons up to age 15 (93).

In the last two decades several studies have investigated the association between diabetes mellitus type 1 and atopic diseases in children, with controversial results.

There is some evidence of a decreased risk of atopy in subjects with T1DM compared to non-diabetic subjects, supporting the Th1-Th2 hypothesis (94–99).

In the multicenter case control study EURODIAB study 2 Study Group, a negative association in children was proved with AD, asthma and rhino-conjunctivitis, respectively (100).

Studies are however contrasting (101–103). Although previous results seem to prove a protective role of atopy from the development of T1DM, other authors suggested a positive association between T1DM and allergic diseases (Table 2).

**Table 2:** Studies showing a positive association between IBD and atopic diseases.

First author and country	Year	Study design	Study group	Found correlation	Results	*P* value	Ref
Lu Z et al. (Taiwan)	2021	Metanalysis of cross-sectional or case–control studies	90,568,121 patients with AD	Atopic dermatitis	Elevated prevalence of CrD in AD patients, with an average OR of 1.66 pooled RR of 1.38 Higher prevalence and incidence of AD in UC patients, with a pooled OR of 1.95 and a pooled RR of 1.49	*p* 0.374–0.426 (CrD), *p* 0.009–0.196 (UC)	(40)
Augustin M. et al. (Germany)	2015	Prevalence data analyses	30,354 children and young adults up to 18 years diagnosed with AD	Atopic dermatitis	1.33 PR of CD and 1.75 PR of UC in patients with and without AD \	*p* < 0.05 (UC), *p* > 0.05 (CrD)	(74)
Schmitt et al. (Germany)	2016	Cohort study	49,847 patients with AD	Atopic dermatitis	Increased risk for incident IBD (CD: RR, 1.34; 95% CI, 1.11–1.61; UC: RR, 1.25; 95% CI, 1.03–1.53)	*p* < 0.05	(75)
Virta L.J. et al. (Finland)	2013	Case-control sudy	595 Finnish children (233 with CD, 362 with UC)	Cow's milk allergy/asthma	Positive association of CMA with CD (OR 1.92, CI 1.09–3.36, *P* < 0.05) and ulcerative colitis (OR 1.71, CI 1.04–2.83, P < 0.05), asthma only with Crohn disease (OR 2.33, CI 1.41–3.86)	*p* < 0.05 (CMA with CrD/UC), *p* < 0.001 (asthma with CrD)	(80)
Virta L.J. et al. (Finland)	2016	Cohort study	7,910 infants with CMA	CMA	Increased incidence of PIBD in patients with CMA (incidence ratio 2.6%–95% CI 1.7–3.8)	*p* > 0.05	(81)
Kappelman M.D. et al. (USA)	2011	Case-control study	737 children with CD, 488 with UC	Asthma/AR/AD	Trend toward an increased prevalence of asthma, eczema, allergic rhinitis	*p* > 0.05	(82)
Kuenzing M. E. et al. (Canada)	2017	Population-based case-control study	3,087 patients with CD and 2,377 with UC (younger than 40 years)	Asthma/AR/AD	Increased odds of incident CD in patients of every age with asthma (OR, 1.45, 95% CI); increased OR (1.49, 95% CI) in UC patients diagnosed at an age of 16 years or less	*p* < 0.0001	(83)

Notably, the strongest associations have been seen with atopic dermatitis and asthma (43, 104): a data analysis from 31 countries in the 13- to 14-year-old age group seemed to demonstrate a common predisposition from environmental and/or genetic factors to type 1 diabetes, wheezing and atopic eczema (105).

On the contrary, no correlation has been observed between rhinitis/rhino-conjunctivitis and T1DM (105).

Few data are available regarding food allergy and T1DM. Still Villanova et al. found low prevalence of sensitization to food allergens in T1DM population (106). An increased risk of developing T1DM was demonstrated in patients who were previously diagnosed with CMA at a mean age of 7.2 months (107). A possible relationship between CMA and DM type 1 could be suggested also from other studies, widely revealing high levels of antibodies against betalactoglobuline and bovine serum albumin in diabetic children (108, 109).

Among different atopic manifestations, these studies mainly focused on the presence of asthma in T1D population (Table 3). Recently Zeng et al.'s metanalysis (110) and Sgrazzutti et al.'s review (111) found a higher risk ratio of developing T1DM in patients who were previously diagnosed with asthma, but not vice versa. In addition, the occurrence of wheezing during the first year of life, which has been considered as a risk factor for later development of asthma, seems to be strongly associated with the risk of *β*-cell autoimmunity (112).

The co-occurrence of both diseases could be explained by defects in immune system response, driven by both peculiar genetic and environmental factors. A promising hypothesis may be the overstimulation of Treg cells: patients with both T1DM and allergy show higher levels of inflammatory cytokines compared to children with only one disease, which also persist despite hypersecretion of anti-inflammatory IL-10, suggesting a functional exhaustion of Tregs (113). Moreover, microbiota and gut barrier's dysfunction seem to play a role in triggering *β*-cells autoimmunity (114). The concomitant presence of T1DM and asthma seems to have implication also for therapy management, with higher insulin doses needed to keep glycemic level in range (115); on the other hand, an increased use of asthma medication in children and adolescent with T1DM in the first year after the diabetes onset than gender- and age-matched diabetes-free controls has been reported (116).

Summing up, in a patient with T1DM, we need to think about the possible coexistence of cow's milk allergy, atopic dermatitis and asthma. In the latter case there is a therapeutic implication as the drugs used in asthma can lead to an increase in insulin doses required to maintain euglycemia.

### Thyroid autoimmune diseases and allergic disorders

Thyroid autoimmune diseases (TAD) are among the most common autoimmune disorders, with a prevalence in the general population of around 5% (118).

TA is known to be associated with chronic urticaria in adult population (119) and in more recent years this correlation has been observed also in children. Urticaria is a skin condition characterized by the appearance of transient (<24 h) red and itchy wheals, of different size, number, and distribution (120); it may present isolated or in association with angioedema. Urticaria is defined chronic (CU) when acute episodes occur (almost) daily for at least 6 consecutive weeks (121). Between 0.5% and 3% of the general population experience CU, with a peak incidence in middle-aged females (122). Concerning childhood, CU is detected at a median age of 6–11 years old, but cases in younger children have also reported (120).

Two types of chronic spontaneous urticaria (CSU) are recognized: autoallergy (also called type I autoimmunity) with IgE autoantibody involvement, and type IIb autoimmunity with IgG autoantibody involvement (123).

Co-occurrence of TAD and CU in children and adolescents has been reported to be lower than in adults, ranging between 4.3 and 26.9%, according to different studies (120, 124, 125).

On the basis of these studies, European Guidelines include screening of TAD in the diagnostic work-up of CU (126). It is also important to periodically screen children diagnosed with CU for TAD over time (120, 124, 125).

Thyroid autoimmune diseases seems to be also associated with atopic dermatitis (AD) (Table 4). Pedullà et al. conducted a study on a pediatric population affected by AD and found that the prevalence of TA was higher than controls, especially in case of IgE-mediated (vs. non-IgE-mediated) AD (127).

Concerning the link between TAD and other allergic diseases, as asthma and allergic rhinitis, up to now, there is no evidence of a possible association between these disorders (128).

The mechanism whereby CU and TAD are associated is not clear yet. It can be hypothesized that the action of the thyroid stimulating hormone on the thyroid gland, when excessive, could lead to inflammation. This may result in disruption of the normal architecture of the gland and subsequent release of antigens, recognized as non-self. A low-grade autoimmune response is then established and immune complexes can activate the complement pathway, leading to C3a and C5a production, and finally mast cell degranulation (129). Another possible explanation is the presence of a specific type of antibodies [IgG autoantibody against the alpha chain of the high affinity IgE receptor (FceRI*α*) on mast cells]able to induce basophildegranulation, found in patients with both CU and Hashimoto thyroiditis (130). In a study by Greaves et al., seven children were tested and three of them presented functional anti- FceRI*α* antibodies (131).

The key message is the one-to-one correlation between autoimmune thyroiditis and chronic urticaria, meaning that the presence of one of the two conditions must lead to screen for the other.

### Juvenile idiopathic arthritis and allergic disorders

Juvenile idiopathic arthritis (JIA) is the most frequent chronic childhood rheumatic disease. Recently, several molecular mechanisms involved in the pathogenesis of the disease have been better defined (132). JIA can be considered an immune-mediated pathology - multifactorial in nature—with simultaneous involvement of both environmental and genetic factors.

Schubert et al. (133) performed a genetic analysis to define a risk profile for asthmatic disease and JIA by studying several genetic polymorphisms on 231 asthmatic children, 86 children with JIA, and 270 controls. They demonstrated an association of IL-4, CTLA4 and TNF-alpha polymorphisms related to asthmatic pathology and/or JIA, with an inverse distribution. The aforementioned genetic data do not allow to define a clear identification of a genetic risk profile, but they strongly suggest that asthma and JIA share the same genetic background and potentially a similar cytokine pattern as well.

Heinzmann et al. (134) recognized the pivotal role played by IL-18 in the regulation of the immunological response as the immune system polarization toward a Th1 and/or Th2 phenotype largely depends on the aforementioned cytokine. Likewise, to our best knowledge, full understanding of IL-18 effects on asthma and JIA is lacking.

Guo et al. (135) suggested that in patients with JIA, atopy coexistence worsens the outcome of arthritis with enthesitis. Indeed, patients with enthesitis-related arthritis (ERA) show involvement of multiple anatomical districts and a higher disease-activity score. The results indicate that atopic patients with ERA requiring biological therapy show a less satisfactory response when compared to the ones without atopy.

Of note, multiple molecular mechanisms are shared between ERA and allergy. In patients with ERA, high levels of IL-17 have been identified, which induces class E antibody recombination. Moreover, in the same patients, increased expression of TLR2 and TLR4—which provide powerful proinflammatory signals—has been recognized. Finally, allergic rhinitis predicts the increased expression of TLR4.

On the other hand, Ozge Avar-Avyn et al. (136) suggest that patients with Th1-driven chronic inflammatory diseases have a reduced frequency of atopic diseases and no effect on disease activity. Similarly, patients with chronic inflammatory diseases on immunosuppressive therapies undergo resolution of allergic symptoms, where present.

Table 5 shows the main studies investigating the association between JIA and atopic disorders.

**Table 3:** Studies investigating the association between T1DM and atopic diseases.

First author and country	Year	Study design	Study group	Found correlation	Results	*P* value	Ref
Lin et al. (Taiwan)	2016	Retrospective cohort study	3,386 patients diagnosed with T1DM, age <18 years	Atopic dermatitis	Overall incidence rate of AD was 1·40-fold (significantly) higher in the T1DM cohort than in the non-T1DM cohort (3·31 vs. 2·35 per 1,000 person years)	*p* 0.01	(104)
Kero et al. (Finland)	2001	Observational epidemiological study	the whole 1,987 birth cohort until 7 years of age	Asthma	Asthma incidence tended to be more common in children with IDDM than in children without IDDM (RR 1.45)	*p* 0.221	(43)
Fsadni et al. (Malta)	2012	Observational epidemiological study	incidence of T1DM from 10- to 14-year-old children (DiaMond Study) and prevalence of wheezing, rhinitis, rhino-conjunctivitis and atopic eczema in 13- to 14-year-old children (ISAAC)	Asthma, rhinitis and AD	Positive correlation between incidence of T1DM with both wheezing and atopic eczema no correlation found with rhinitis or rhino-conjunctivitis.	*p* 0.009 (wheezing), *p *< 0.01 (AD)	(105)
Klamt et al. (Germany)	2015	Prospective case-control study	94 children and adolescents with T1DM, aged between 3 and 21 years	IgE-mediated allergies	Significantly higher risk for a positive personal history for allergic symptoms in children with T1DM (OR 1.88)	*p* 0.026	(117)
Villa-nova et al. (Brazil)	2014	Descriptive cross-sectional study	96 patients with DM1, aged between 3 and 18 years	Asthma, rhinitis, AD and sensitization on SPT to aero- and food allergens	Prevalence values of rhinitis, asthma and atopic eczema (isolated or associated) of 68.0%, 59.1% and 44.4%, respectively. 48% patients sensitized on SPT.	N/A	(106)
Lamminsalo et al. (Finland)	2021	Register-based case-cohort study	7,745 children diagnosed with T1DM before 16 years of age	Cow milk allergy	Increased risk of developing T1DM in children with CMA in fully adjusted model (HR = 1.17; 95% CI 1.02–1.34)	*p* < 0.05	(107)
Zeng et al. (China)	2022	Meta-analysis on 22 observational studies	25,578 patients with T1D (adults and children)	Asthma	No apparent connectivity between asthma and T1D (crude OR 1.07); positive association between T1D and asthma in meta-analysis of 6 studies with adjusted OR (aOR 1.15) and in meta-analysis of cohort studies (aOR 1.15 and pooled cOR 1.27).	*p* < 0.05	(110)
Wahlberg at al. (Sweden)	2011	Population-based prospective cohort study	7,208 unselected 2.5-yr-old children	Wheezing	Strong association between occurrence of wheezing in the first year of life and *β*-cell autoimmunity at the age of 2.5 year (OR 10.7 for both GADA and IA-2A)	*p* 0.000	(112)
Hörtenhuber et al. (Germany and Austria)	2018	Prospective multicenter observational cohort study	51 926 patients with T1D (<20 years)	Asthma	Higher insulin doses needed in patients with asthma and T1DM (0.88 ± 0.3 vs 0.84 ± 0.3 U/kg,)	*p* < 0.01	(115)
Ahmadizar et al. (Netherlands)	2016	Population-based cohort study	915 patients younger than 19 years with at least 2 insulin prescriptions (1999–2009)	Asthma	Significantly higher prevalence rate of asthma medication use in the T1DM cohort (23.2%) than the reference cohort (18.3%) after the onset of diabetes	*p* < 0.01 (0–4 year) *p* 0.02 (10–14 year)	(116)
Stene LC et al. (Norway)	2004	Population-based case-control study	545 cases of childhood-onset type 1 diabetes	AD, allergic rhino-conjunctivitis and asthma	Inverse association of AD with risk of type 1 diabetes, odds ratio = 0.55 (95% confidence interval 0.35–0.87), no significant association with allergic RC and asthma	*p* < 0.05	(94)
Stene LC et al. (Norway)	2010	Case-control study	339 incident cases of T1DM from Norwegian childhood diabetes registry	AD	Lower risk of T1DM development in AD children (OR 0.61, 95% CI 0.40–0.95)	*p* < 0.05	(95)
Rosenbauer J et al. (Germany)	2003	Population-based case-control study	760 cases newly-diagnosed with Type 1 diabetes under five years of ag	AD, allergic rhino-conjunctivitis and asthma	Reduced risk of T1DM in AD children, adjusted OR 0.71 (95% CI 0.53–0.96), no significant association with hay fever and asthma	*p* < 0.05	(96)
Thomsen FS et al. (Denmark)	2011	Population-based case-control study (co-twin control analysis)	54.530 Danish twin subjects, 3–71 years of age	AD, allergic rhino-conjunctivitis and asthma	Lower risk od AD in DM children, OR = 0.23 (0.07–0.71); no significant association with hay fever and asthma	*p* < 0.011	(97)
Mattila PS et al. (Finland)	2002	Case-control study	306 probands with childhood type 1 diabetes	Asthma, animal dust and pollen allergy	Risk of DM inversely associated with asthma (odds ratio 0.49 [95% CI 0.24–1.00]), allergy to animal dust (0.67 [0.45–0.99]), and to a lesser degree to pollen (0.74 [0.51–1.07])	animal dust *p* 0.045, asthma *p* 0.0505	(98)
Meerwaldt et al. (Netherlands)	2002	Case-control study	555 children with DM	Asthma, AD, allergic RC	Lower prevalence of asthma OR 0.796, 95% CI 0.408–1.554), hayfever (OR 0.642, 95% CI 0.369–1.118) and AD symptoms (OR 0.693, 95% CI 0.430–1.115) in DM patients	*p* > 0.05	(99)
The EURODIAB Substudy 2 Study Group	2000	Population-based case-control study	1,028 members of case group (T1DM)	AD, allergic rhino-conjunctivitis and asthma	Combined OR significantly decreased, if considered any of 3 atopic dsorders (OR 0.82; 95% CI 0.68, 0.98, *P* = .03) with evidence of heterogeneity among centers. Consistent risk reduction associated with asthma in all centers (R 0.70. CI 0.54–0.91)	*p* 0.03 (any atopic disorder), *p* 0.008 (asthma)	(100)

**Table 4:** Studies investigating the association between TAD and atopic diseases.

First author and country	Year	Study design	Study group	Atopic disorder	Results	*P* value	Ref
Pedullà et al. (Italy)	2014	Case-control study	217 children (147 affected by AD, 70 control)	Atopic dermatitis	Significant association between frequency of atopy and thyroid autoimmunity; in particular, prevalence of thyroid autoimmunity was higher in children affected by AD (9.52% vs. 0)	*p* < 0.05	(127)
Pedullà et al. (Italy)	2016	Case-control study	324 children referred to the Pediatric Department for skin diseases	Atopic dermatitis	The authors observed a significant association between atopy and thyroid autoimmunity (TA) in atopic children with skin disease. This association was confirmed as significant in atopic children affected by atopic dermatitis (11.5% vs. 2.7%, OR 4.68 [1.02–21.38]).	*p* = 0.03	(125)
Kilic et al. (Turkey)	2010	Observational study	40 children affected by chronic urticaria	Chronic urticaria	Higher prevalence of thyroid autoimmunity (14.8%) in children affected by chronic idiopathic urticaria with respect to general pediatric population	N/A	(120)
Levy et al. (Israel)	2003	Observational study	187 children affected by chronic urticaria	Chronic urticaria	The prevalence of thyroid autoimmunity in children with CU (4.3%) was much lower than that in adult series of chronic urticaria, but higher than the prevalence reported for age matched children (0.35–1.6%)	N/A	(124)
Ademhan Tural et al. (Turkey)	2021	Case-control study	600 children (300 affect by autoimmune thyroiditis, 300 control)	Allergic rhinitis, asthma	No significant association between AR/asthma and thyroid autoimmunity	Not statistically significant	(128)

AD, atopic dermatitis; AR, allergic rhinitis; CU, chronic urticaria; OR, odds ratios.

**Table 5:** Studies investigating the association between JIA and atopic diseases.

First author and country	Year	Study design	Study group	Atopic disorder	Results	*P* value	Ref
Karsh et al. (Canada)	2005	Retrospective case-control study	General Canadian population (data were taken from the Canadian Community Health Survey (CCHS) conducted by Statistics Canada in 2000–2001. The target population included household residents aged 12 years or older)	Allergies (food and non-food allergies)	Allergy history was positively related to the prevalence of rheumatoid arthritis both in women (OR: 1.57 [1.43, 1.73]) and in men (OR: 1.55, [1.36, 1.77])	N/A	(138)
Avar-Aydin et al. (Turkey)	2021	Prospective case-control study	99 patients diagnosed with JIA aged between 2 and 16 years were enrolled in a prospective study as JIA group. An age and sex-matched group of 128 children without any chronic or acute inflammatory diseases were recruited as the control group	Asthma, allergic rhinitis	Despite similar allergy risk factors, the frequencies of asthma and allergic rhinitis were lower in JIA group (4 vs. 12.5% and 1 vs. 7.8% respectively)	*p* < 0.02	(136)

OR, odds ratios; JIA, juvenile idiopathic arthritis.

Ramirez-Bello et al. (137) have stated that the FCLR3 3C, 5C, and 6A alleles are protective factors for the onset of JIA and asthma. FCLR3 has been particularly studied as protective or modifying factor for some diseases, as RA and SLE. This study is not conclusive and other genes and cohorts require genotyping in order to highlight the different effects of FCRL3 variants on immune diseases.

Karsch et al. (138) reported that the cumulative incidence of asthma is significantly correlated with the concomitant presence of CD and rheumatoid arthritis. According to Bach et al, the observed increase in allergic and autoimmune diseases is due to a reduction in infectious diseases which, by means of IL10 and TGF-beta, can inhibit Th1 and Th2 responses. Jimenez-Morales et al. (139) have confirmed the association between a specific TNF 308 A allele and pediatric inflammatory and immune diseases in the Latin American population.

Heinzmann et al. (134) have focused on the same gene variant in populations with different chronic inflammatory diseases such as bronchial asthma, atopy, and JIA. They showed that the Arg110Gln variant is much more common in asthmatic patients. This genetic variant is responsible for an increased production of IL-13 which is a potent inducer of the Th2 response. In this sense, immunological phenotypes with high serum IL-13 levels may have a greater risk of developing allergic diseases and a lower risk of Th1-related immune diseases.

Chi-Wu et al. (140) highlighted the link between AD and SLE (Systemic lupus erythematosus) sharing an immune dysregulation characterized by functional/quantitative reduction of CD4 + cd25 + Foxp3 + cells. They stated that female patients with AD under the age of 18 have a high risk of developing SLE. This association is also confirmed by the retrospective cohort study of Wei et al. (141) that revealed a significantly increased incidence of SLE in children with AD, regardless of sex.

To date, an exhaustive analysis of the pathogenetic factors underlying JIA is not yet known. Barrier defects and the breakdown of immunological tolerance could be involved in the pathophysiology.

An explanatory model may be represented by the formation of anti-citrulline antibodies in JIA: the citrullinated proteins produced in the inflamed synovium bypass the mechanism of thymic selection and induce the abnormal activation of self-reactive T lymphocytes (142).

The evidence regarding the link between JIA and atopy is weak and inconsistent. What is known is that the two conditions share the same cytokine pattern and genetic background;, by a clinical point of view, the strongest association is that patients affected by ERA in biological therapy have a less satisfactory response if they are atopic too.

### Limitations

Our review presents some limitations. First, the study has considered associations between allergy and most common autoimmune disease where data are more abundant. Data from other autoimmune disease such as autoimmune hepatitis, Addison's disease, juvenile dermatomyositis, lupus erythematosus, scleroderma, have not been included in the review, since the data are more scarce. Second, the search strategy of the review only has focused on scientific research since 2,000 and restricted to the pediatric age. Furthermore, many studies are retrospective cohort studies and the timing of occurrence of the disease is not well understood; both of them limit the quality of the evidence and hamper to draw conclusions.

Further powered-prospective studies are warranted to investigate the association among autoimmune and allergic diseases both in children and adults.

## Conclusions

Allergic diseases and autoimmune disorders are often associated in children, proving that Th1 and Th2 responses may coexist, as result of shared immune dysregulation (Figure 1).

B reg and T reg cells play an essential role, given their role in modulating the inflammation's inhibition. Their imbalance leads to the persistence of an inflammatory stimulus, which in turn causes dysregulation of both innate and antibody immunity.

As a consequence, clinicians should pay particular attention to the presence or the development of autoimmune diseases in patients with allergic disorders, particularly atopic dermatitis and asthma, celiac disease and type 1 diabetes and vice versa.

Further studies are warranted to better clarify the common pathogenetic mechanisms underlying these disorders and their temporal association, in order to ameliorate affected patients’ management, quality of life and prognosis.
